# Antimycobacterial Activities of Novel 5-(1H-1,2,3-Triazolyl)Methyl Oxazolidinones

**DOI:** 10.1155/2012/289136

**Published:** 2012-04-11

**Authors:** Oludotun Adebayo Phillips, Edet Ekpenyong Udo, Reny Varghese

**Affiliations:** ^1^Department of Pharmaceutical Chemistry, Faculty of Pharmacy, Kuwait University, P.O. Box 24923, Safat 13110, Kuwait; ^2^Department of Microbiology, Faculty of Medicine, Kuwait University, P.O. Box 24923, Safat 13110, Kuwait

## Abstract

The antibacterial activities of a series of triazolyl oxazolidinones against *Mycobacterium tuberculosis* strain *in vitro* and *in vivo* in a mice model are presented. Most active compounds were noncytotoxic against VERO cells with acceptable selectivity indexes (SI) as measures of compound tolerability. Structure activity relationships (SARs) revealed that analogs with alkylcarbonyl (IC_90_: < 0.2 to 0.422 **μ**g/mL) and arylcarbonyl (IC_90_: < 0.2 to 2.103 **μ**g/mL) groups at the piperazine 4N-position-displayed potent antimycobacterium activities, comparable to the methanesulfonyl (IC_90_: < 0.2 **μ**g/mL) analog, linezolid (IC_90_: < 0.2 **μ**g/mL), and isoniazid (IC_90_: < 0.034 **μ**g/mL). The furanylcarbonyl derivative also displayed potent activity, while the arylsulfonyl analogs were inactive. Of the triazolyl oxazolidinones, the morpholino (PH-27) derivative with medium bioavailability in plasma was most active *in vivo*, but relatively less efficacious than isoniazid.

## 1. Introduction

Tuberculosis (TB) caused by *Mycobacterium tuberculosis*, a contagious and fatal disease, is considered a global epidemic and a major threat to public health. TB is becoming more prevalent in the world today than any other time in human history. It has been estimated that over a third of the world's population is infected with the TB bacilli, with 9.4 million new cases and nearly 1.7 million deaths in 2009 [1, 2]. Most infected people harbor latent TB infection (LTBI), and people with HIV/AIDS and compromised immune system are more likely to develop the disease. TB continues to be a leading cause of death in HIV/AIDS patients, forming a lethal combination.

**Figure 1:**
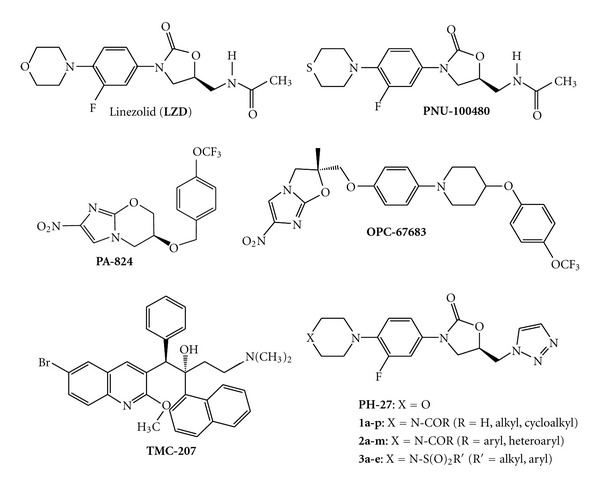
Chemical structure of oxazolidinone antibacterial agents and novel antimycobacterial agents.

A number of drugs, namely, isoniazid, rifampicin, ethambutol, and pyrazinamide are often administered over a prolonged period of time and may lead to the development of resistant strains due to patient-poor compliance among other factors. The development and spread of resistant *M. tuberculosis, *pose a vital challenge to the control of TB world-wide. In recent years, the emergence and spread of resistant *M. tuberculosis* strains has fuelled the TB epidemic by making it more difficult to treat. Multidrug-resistant (MDR) TB, which is resistant to the first line anti-TB agents, namely, isoniazid and rifampicin are increasing with >500,000/yr new cases of MDR-TB worldwide [3, 4]. Extensively drug-resistant (XDR) TB, resistant to first line anti-TB agents, namely, isoniazid, rifampicin, fluoroquinolones, and one of three injectable drugs, such as capreomycin, kanamycin, or amikacin is awfully difficult to treat and is considered a worldwide threat to TB control [3, 4]. These statistics serve as impetus for development of more effective and safer anti-TB drugs [5]. For more than 40 years, there has been a drought of new anti-TB drugs. However, more recently, there are increasing reports of newer agents demonstrating activity against drug-resistant *M. tuberculosis* strains [5–8]. Among these new agents are the oxazolidinones (linezolid—**LZD**; and **PNU-100480**; Figure 1), diarylquinoline (**TMC-207**; Figure 1), the nitroimidazole-oxazole (**OPC-67683**; Figure 1), the nitroimidazo-oxazine (**PA-824**, Figure 1), and some quinolone class of compounds. Linezolid (**LZD**; Figure 1), a prototypical oxazolidinone used in the clinic to treat gram-positive bacterial infections, the thiomorpholino derivative of **LZD** (**PNU-100480**; Figure 1), and others in this class, have demonstrated activity against susceptible and drug-resistant *M. tuberculosis*. Our laboratories have been interested in the synthesis of triazolylmethyl oxazolidinones of general structures **PH-27**, **1**, **2**, and **3** (Figure 1) with antibacterial activity [9–12]. Studies from other laboratories and ours have shown that the triazolyl oxazolidinones have potent activity against gram-positive bacterial species [9–14], comparable or superior to linezolid *in vitro*, thus affirming the bioisosteric replacement of the C5 acetamido functional group by the triazolyl moiety. On the basis of the potent antibacterial activities of the triazolyl oxazolidinones and the demonstrated antimycobacterium activities of representative oxazolidinones, namely, **LZD** and **PNU-100480**, we evaluated the anti*Mycobacterium tuberculosis* activity of selected novel triazolylmethyl oxazolidinones synthesized from our laboratories. Therefore, this study outlines the *in vitro* and *in vivo* antimycobacterial activity of selected triazolylmethyl oxazolidinones and to establish observable qualitative structure-activity relationships.

## 2. Materials and Methods

### 2.1. Synthesis of Compounds

The preparation of the compounds **1a-p**, **2a-m**, and **3a-e** has been described previously [9–12]. The reference antituberculosis agents isoniazid (**INH**) and linezolid (**LZD**) were provided by the Tuberculosis Antimicrobial Acquisition and Coordinating Facility (TAACF), Southern Research Institute, Birmingham, AL., USA.

### 2.2. Antituberculosis Susceptibility Testing

 The *in vitro* antitubercular testing was determined according to the protocols of the Tuberculosis Antimicrobial Acquisition and Coordinating Facility (TAACF), USA [15, 16]. The activity of all compounds against replicating *M. tuberculosis* H37Rv (ATCC 27294, American Type Culture Collection, Rockville, MD, USA) was performed in BACTEC 12B medium using a fluorescence readout in the Microplate Alamar Blue Assay (MABA) according to the TAACF initial primary screen assays. Compounds were dissolved in 80% DMSO or 60% EtOH in water or H_2_O and tested in ten 2-fold dilutions, ranging from 0.19 to 100 *μ*g/mL; the IC_90_ (*μ*g/mL) values defined as “inhibitory concentration” effecting a reduction in fluorescence of 90% relative to the controls. The values were determined from dose-response curves, and compounds with IC_90_ ≤ 10 *μ*g/mL were considered active for anti-tubercular activity.

### 2.3. Mammalian VERO Cell Cytotoxicity (CC_50_) Assay

The compounds were screened to assess toxicity to African green monkey kidney (VERO) cells using the Promage's Cell Titer Glo Luminescent Cell Viability assay by TAACF. The assay returns a CC_50_ value, which allows a selectivity index (SI: ratio of CC_50_/IC_90_) to be calculated. Compounds with SI value ≥ 10 are considered safe for further screening.

### 2.4. Preliminary *In Vivo* Bioavailability Testing (BioAssay)

All *in vivo* assays/evaluations were determined according to the TAACF protocols [15]. This assay was used to estimate drug levels in mice at specific time points after oral dosing, using the inhibition of growth of *M. tuberculosis* H37Rv as an indicator for drug activity. Selected compounds were orally dosed (300 mg/kg in 0.5% methyl cellulose) in three C57BL/6 mice and the animals were bled by nicking the lateral vein at 30 mins and 2.5 hrs, respectively, to collect blood samples. The blood samples were allowed to clot and centrifuged to collect serum, which were serially diluted and added to 96-well assay plates containing *M. tuberculosis* H37Rv (10^4^ bacterial) on 100 *μ*L 7H9 broth with 10% serum. Each assay also contains lanes of wells with drugs of known concentrations, with or without 10% mouse serum. Inhibition of bacterial growth was determined by optical density (*λ* = 600 nm) measurements every 3-4 days up to 14 days. The results were further confirmed by visual inspection at 10 days. Inhibition of bacterial growth in the assay (<50% of control without drugs) indicates sufficiently high concentration of compound in blood and hence an acceptable bioavailability.

### 2.5. *In Vivo* Efficacy Gamma Knock-Out (GKO) Mouse Model

 Interferon gamma knockout female mice C57BL/6 IFN-KO (**n** = 5) unable to control an infection of *M. tuberculosis* were infected with *M. tuberculosis* ATCC 35801 (Erdman strain) by aerosol infection utilizing the Glass-Col Inhalation Exposure System. Treatment was started on day 13 after infection for 9 consecutive daily treatments until day 21. Test compounds were dissolved in 0.5% methyl cellulose and administered via oral gavage. An isoniazid (**INH**) control group, administered via oral gavage at 25 mg/Kg/day (H_2_O), was included in each study. Mice were sacrificed on day 22 postinfection, and bacterial loads in the lungs and spleen were determined [15]. The Log10 CFU reduction values > 0.30 generally indicate activity.

## 3. Results and Discussion

The main goals of this study included evaluating the antimycobacterial activities of a series of triazolyl oxazolidinone derivatives **1a-p** and **2a-m** and **3a-e**, *in vitro* and *in vivo,* and explore recognizable structure-activity relationships (SARs) around the phenyloxazolidinone moiety. The synthesis of the compounds was described previously [9–12]. The results from the *in vitro* and *in vivo* (mice model) studies, presented in the Tables 1–5, were determined according to TAACF protocols [15, 16]. Data from *in vitro* antimycobacterium and VERO cytoctoxicity assays are presented in Tables 1 and 2. From these data most of the compounds displayed potent to lack of activity against *M. tuberculosis* H37Rv strain with IC_90_ values in the range of <0.2 to >100 *μ*g/mL, while the reference compounds linezolid (**LZD**) and isoniazid (**INH**) showed IC_90_ values of <0.2 and 0.034 *μ*g/mL, respectively. The 4N-acylpiperazinyl derivatives displayed potent activity (IC_90_ range of >0.2–0.422 *μ*g/mL, Table 1), in comparison to the 4N-aryl-carbonylpiperazinyl derivatives, which were relatively less active with IC_90_ in the range of <0.2–2.103 *μ*g/mL. On the other hand, the 4N-arylsulfonyl derivatives were devoid of antimycobacterium activity (IC_90_ range of 5.469–100 *μ*g/mL), while the methane-sulfonylpiperazinyl derivative **3a** showed potent activity with IC_90_ value of <0.2 *μ*g/mL. Although positive correlations between log *P* values and antimycobacterium activity have been demonstrated by previous other studies [17], however, such correlations could not be drawn in the present study since the MIC end-points were not determined. The *C*log⁡*P* values of the compounds were estimated using ChemDraw Ultra 8.0.

In this study, interpretation of the *in vitro* assays (IC_90_ and VERO cell cytotoxicity: CC_50_) data assisted in selection of compounds for further testing *in vivo*. Hence, data from the selectivity index (SI ratio = CC_50_/IC_90_, Tables 1 and 2) further indicated that most of the active compounds also displayed acceptable safety and therapeutic index profiles, represented by their SI ranges of >21 to >250. According to the TAACF criteria, compounds with SI value ≥ 10 are considered safe for further screening. In this regard, nine triazolyl oxazolidinones (**1g**,** 1i**,** 1o**,** 2b**,** 2j**,** 2k**,** 2l**,** 3a**, and **PH-27**) along with **LZD** and **INH** as reference compounds were selected for preliminary bioavailability study. This bioassay was used to estimate the levels of the drug in mice at specific times after oral dosing. From the results presented in Table 3, the reference compounds **LZD** and **INH** displayed high concentrations (118.4 and 4.48 *μ*g/mL) of the drugs in mice serum, suggesting high bioavailability of the drugs as demonstrated by the activity against *M. tuberculosis* H37Rv in 5 or 6 wells. While three of the triazolyl oxazolidinones, namely, **1g** (4N-isobutyryl), **2l** (4N-nicotinoyl), and **PH-27** (morpholino) displayed low to medium concentrations in mice, indicating low-to-medium bioavailability of drugs as demonstrated by activity in only 1 or 2 and 3 or 4 wells of the bioassay, respectively. In addition, the most lipophilic compound, the 4N-undecanoyl derivative **1o **(*C*log⁡*P* = 3.5568) demonstrated 10 times higher MIC in the presence of serum suggesting that this compound probably has higher protein binding. Although all the other 5 compounds tested also showed relatively insignificant protein binding (Table 3), they also demonstrated no bioavailability similar to that of 4N-undecanoyl derivative **1o**. The probable reason(s) for this lack of bioavailability of these other 5 compounds could be due to rapid metabolism and/or poor permeability of the compounds.

Finally, the *in vivo* antimycobacterial activities of the compounds that displayed bioavailability of low to high concentrations were evaluated against gamma knock-out (GKO) C57BL/6 female mice model, which are unable to control aerosol infection with the Erdman strains ATCC 35801. Treatment was initiated 13 days after infection by orally administering the drugs and treatment continued for 9 consecutive days until 21 days. The results of this *in vivo* study are presented in Tables 4 and 5 following sacrifice of the animals. As can be seen in Table 4, the triazolyl oxazolidinones **1g (306027)** and **PH-27 (306006)** demonstrated efficacy comparable to **INH** in mice lungs and spleen, while **2l (306019)** were less efficacious. Generally, the triazolyl compounds were more effective in lungs. Furthermore, mice treated with the triazolyl oxazolidinones presented with distended large intestine. The morpholino derivative **PH-27** was the most active of the triazolyl oxazolidinones tested providing 1.44 log⁡ CFU reduction in the lung and 1.74 log⁡ reduction in the spleen. While the 4N-isobutyryl derivative **1g** showed 0.78 log⁡ CFU reduction in the lungs, which was statistically significant, but no statistically significant activity was noted in the spleen. On the contrary, the 4N-nicotinoyl derivative **2l** did not show statistically significant log⁡ CFU reduction in either lung or spleen with 0.39 and 0.14 log⁡ CFU (Table 5), respectively. However, the reference compound **INH** provided 3.29 log⁡ and 4.34 log⁡ CFU reduction in lungs and spleen, respectively.

The main reason for the significant differences in the *in vivo* efficacy of these three derivatives 4N-isobutyryl **1g**, 4N-nicotinoyl **2l**, and morpholino **PH-27** is not clear, apart from the obvious differences in the preliminary bioavailability results. Although the bioavailability studies of the efficacious compounds are very preliminary in nature, the efficacy results seem to go well in hand with the reported oral exposure of the compounds. Previous studies from our laboratory have shown that these class of compounds are relatively stable in plasma at physiologic conditions and may bind to plasma protein [18]. In particular, the most probable reasons for lack of bioavailability of the six triazolyl oxazolidinones **1i**,** 1o**,** 2b**,** 2j**,** 2k**, and** 3a** may be due to rapid *in vivo* metabolism and/or poor permeability upon oral administration. Further investigations on these class of compounds and other derivatives including their monoamine oxidase inhibitory activities are ongoing in our laboratories.

## 4. Conclusion

This study discloses the *in vitro* and *in vivo *antimycobacterium activity of a series of triazolyl oxazolidinones. Most of the compounds displayed potent *in vitro* activity against *M. tuberculosis* H37Rv, but this *in vitro* potency was not strongly reflected at the same level *in vivo*. This could be due to the low bioavailability of the compounds *in vivo*, however, the precise reason for this is unresolved. In conclusion, these derivatives may serve as templates for further modifications to attain more effective antimycobacterial compounds.

## Figures and Tables

**Table 1:** *In vitro* antimycobacterium activity of 4N-acylpiperazinyl oxazolidinones against *M. tuberculosis* H37Rv.

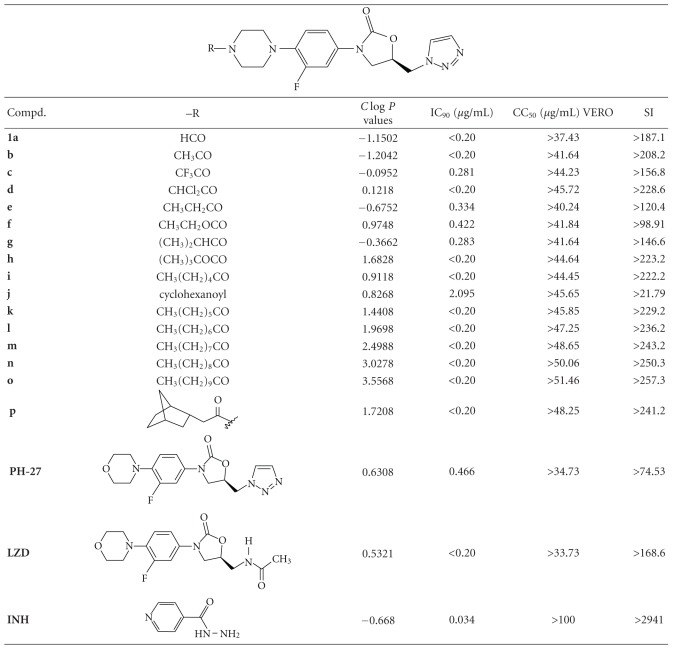

**Table 2:** *In vitro* antimycobacterium activity of 4N-arylcarbonyl- and 4N-arylsulfonyl-piperazinyl oxazolidinones against *M. tuberculosis* H37Rv.

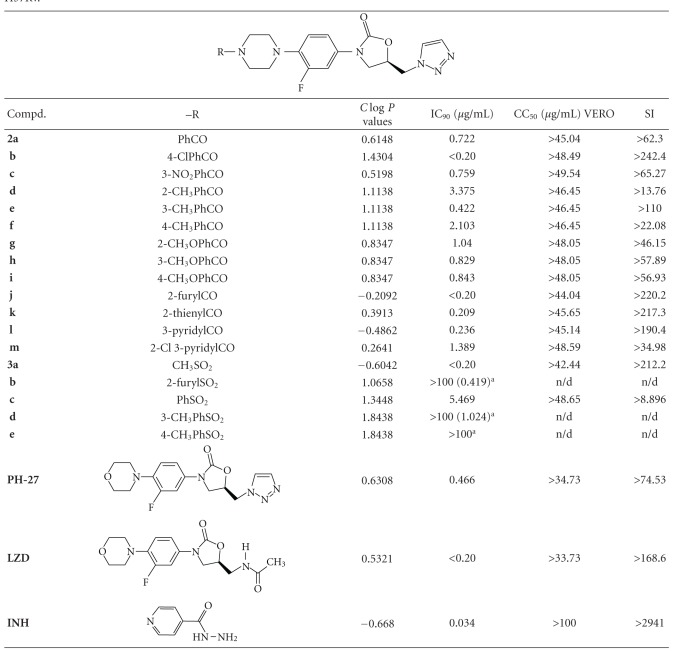

^a^Concentration that inhibited 50% growth of *M. tb*. (IC_50_).

**Table 3:** *In vivo *bioavailability of selected oxazolidinones.

Compd.	~ MIC (*μ*g/mL) − serum	~ MIC (*μ*g/mL) + serum	Dilution factor^a^	~ Compd in serum (*μ*g/mL)	Bioavailability^b^
**1g**	1.1	3.3	1 : 20	22	low
**1i**	3.3	3.3	0	0	none
**1o**	0.37	3.3	0	0	none
**2b**	3.3	3.3	0	0	none
**2j**	1.1	1.1	0	0	none
**2k**	1.1	1.1	0	0	none
**2l**	1.1	0.37	1 : 10	11	low
**3a**	0.37	1.1	0	0	none
**PH-27**	1.1	0.37	1 : 80	88	medium
**LZD**	0.37	0.37	1 : 320	118.4	high
**INH**	0.014	0.041	1 : 320	4.48	high

^a^Dilution factor represents the last dilution step of the serum samples in which drug activity was still observed in the bioassay. (Drug levels in mouse serum are estimated by multiplying the dilution factor by the MIC value of the drug in absence of serum).

^b^Rating of bioavailability: low: activity of drug can be seen in 1 or 2 wells of the bioassay; medium: activity of drug can be seen in 3 or 4 wells of the bioassay; high: activity of drug can be seen in 5 or 6 wells.

**Table 4:** *In vivo* efficacy and observation at time of sacrifice.

Compd.	Lungs	Spleen	Other remarks
Control	+++	6 enlarged; 1 very large	2 with enlarged lymph nodes
** INH **	+	1 slightly enlarged; 4 normal	n/a
** 2l (306019) **	++	1 enlarged; 4 slightly enlarged	Large intestine distended full of feces and air
** 1g (306027) **	+	1 slightly enlarged; 4 normal	Large intestine distended full of feces and air
** PH-27 (306006) **	+	normal	Large intestine distended full of feces and air

+++; highly infected. ++/+; hardly any infected. n/a; not applicable.

**Table 5:** *In vivo* efficacy log10 CFU reduction in bacterial load in lungs and spleen.

Test group	Organ	Dose mg/Kg/dy	Mean ± SEM CFU	Log10 CFU reduction	Activity
Untreated **d13**	Lung	n/a	6.90 ± 0.11	n/a	n/a
	Spleen		4.75 ± 0.21		

Untreated **d22**	Lung	n/a	8.06 ± 0.11	n/a	n/a
	Spleen		6.38 ± 0.07		

** INH **	Lung	25	4.77 ± 0.12	3.29	Active
	Spleen		2.04 ± 0.38	4.34	Active

** 2l (306019) **	Lung	150	7.67 ± 0.22	0.39	Inactive^a^
	Spleen		6.24 ± 0.06	0.14	Inactive^a^

** 1g (306027) **	Lung	300	7.28 ± 0.14	0.78	Slightly active
	Spleen		5.95 ± 0.09	0.43	Inactive^a^

** PH-27 (306006) **	Lung	150	6.62 ± 0.08	1.44	Active
	Spleen		4.64 ± 0.21	1.74	Active

^a^306027: slightly active in lung, but activity in spleen was not statistically significant.
